# National Association, No. 2

**Published:** 1884-06

**Authors:** Henry W. Morgan

**Affiliations:** Nashville, Tenn.


### National Association No. 2.

Editor Register—*Dear Sir:* Permit me, through the Register, to ask a few questions?

Will the proposed organization of another Association of dentists under the name of the "National Dental Association," tend to elevate the regard of the profession abroad? Is this an opportune time for such an attempt?

The argument has been put forth that we have no "truly national" representative body of dentists, and that the formation of an association to hold its meetings annually at Washington, with a museum and library, "would give to the nation, claiming to lead the world in the science and art of dentistry, a representative body to speak forth with authority its aims and attainments."

This is claimed as an "original idea." Well, that part of it in which the word "annually" occurs may be, but some of us remember an association with all these features which was to meet alternately at Washington and elsewhere, organized in New York in 1880, and which has held but two meetings since, one of these in Washington with hardly a corporal's guard.

Mark you a representative body, where were the representatives?

Now, what has become of the American and Southern Dental Associations?

The former has done good work, although it has "sat upon" some and "trod upon the toes" of others. Who were the men who commanded the attention and admiration in the Medical Congress in 1880 in the section devoted to the "Teeth and Their Diseases?" Members of the American Dental Association—many of them among its original projectors and organizers.

Whose papers are copied into foreign journals?

Is this association a representative body? At its last meeting, held at Niagara, in August, 1883,* there were twenty-six societies represented by fifty-five representatives. In other words, more than half of the number present were delegates.

The Southern Association for a while seemed to be on the wane on account of lack of interest and proper management, but it is now in the ascendency, having held the best meeting it has ever had at Atlanta last year.

*There were over one hundred members present, representing twenty states, and there are members in almost every State in the Union. We have not had time to write to the Secretary for figures.

With these two associations, with bright hopes for long and useful lives, composed of as good material as can be drawn into *any* other organization, what need have we for another representative body just now? To create more offices for the less fortunate?

Have either of the before named associations ever refused to listen to the learned or to reward and honor the deserving?

The proposed "National" is to give to each State "six delegates," thus giving to Rhode Island, with barely a hundred dentists, the same representation as New York, with nearly two thousand. Will this be a *representative* body? In some States where the number in the profession is great, and there are necessarily more than one association, all except some favored society are to be excluded.

Let the first "National" die and be decently buried before the organization of the second. And instead of trying to create a new association to supplant or consolidate all those already in existence, let the profession rally around those doing good and faithful work.

We should be a little more concerned about our home reputation, and let all our efforts be to elevate that, then the estimate to be placed upon it by those abroad will need no looking after.

HENRY W. MORGAN,
Nashville, Tenn.

